# Peroxisome proliferator-activated receptor alpha plays a crucial role in behavioral repetition and cognitive flexibility in mice

**DOI:** 10.1016/j.molmet.2015.04.005

**Published:** 2015-05-02

**Authors:** Giuseppe D'Agostino, Claudia Cristiano, David J. Lyons, Rita Citraro, Emilio Russo, Carmen Avagliano, Roberto Russo, Giuseppina Mattace Raso, Rosaria Meli, Giovambattista De Sarro, Lora K. Heisler, Antonio Calignano

**Affiliations:** 1 Department of Pharmacy, University of Naples Federico II, Naples, Italy; 2 Rowett Institute of Nutrition and Health, University of Aberdeen, Aberdeen, United Kingdom; 3 Drug Discovery and Development, Italian Institute of Technology, Genoa, Italy; 4 Science of Health Department, School of Medicine, University “Magna Graecia” of Catanzaro, Catanzaro, Italy

**Keywords:** Lipids, Nuclear receptor, Ketamine, Memory, Neurodevelopmental disorders, Stereotyped behavior

## Abstract

**Background/objectives:**

Nuclear peroxisome proliferator activated receptor-α (PPAR-α) plays a fundamental role in the regulation of lipid homeostasis and is the target of medications used to treat dyslipidemia. However, little is known about the role of PPAR-α in mouse behavior.

**Methods:**

To investigate the function of *Ppar-α* in cognitive functions, a behavioral phenotype analysis of mice with a targeted genetic disruption of *Ppar*-α was performed in combination with neuroanatomical, biochemical and pharmacological manipulations. The therapeutic exploitability of PPAR-α was probed in mice using a pharmacological model of psychosis and a genetic model (*BTBR T* + *tf/J*) exhibiting a high rate of repetitive behavior.

**Results:**

An unexpected role for brain *Ppar*-α in the regulation of cognitive behavior in mice was revealed. Specifically, we observed that *Ppar*-α genetic perturbation promotes rewiring of cortical and hippocampal regions and a behavioral phenotype of cognitive inflexibility, perseveration and blunted responses to psychomimetic drugs. Furthermore, we demonstrate that the antipsychotic and autism spectrum disorder (ASD) medication risperidone ameliorates the behavioral profile of *Ppar*-α deficient mice. Importantly, we reveal that pharmacological PPAR-α agonist treatment in mice improves behavior in a pharmacological model of ketamine-induced behavioral dysinhibition and repetitive behavior in *BTBR T* + *tf/J* mice.

**Conclusion:**

Our data indicate that *Ppar*-α is required for normal cognitive function and that pharmacological stimulation of PPAR-α improves cognitive function in pharmacological and genetic models of impaired cognitive function in mice. These results thereby reveal an unforeseen therapeutic application for a class of drugs currently in human use.

## Highlights

• The lipid nuclear receptor PPAR-α is required for normal cognitive function in mice.
• Genetic ablation of *Ppar-α* promotes repetitive behavior and cognitive inflexibility in mice.
• Pharmacological stimulation of PPAR-α improves behaviors in models of impaired cognition and enhanced repetitive behavior.
• Our findings suggest *Ppar-α* as a possible point of commonality between behavioral and metabolic dysfunctions.

## 1 Introduction

Dyslipidemia, a dysregulation of lipid content in the blood, is a leading risk factor for cardiovascular disease (CVD). Dyslipidemia is treated with fibrates, which act as agonists of the nuclear receptor peroxisome proliferator-activated receptor-α (PPAR-α) [1,2]. This protein is one of three PPAR isotypes, PPAR-α, PPAR-γ, and PPAR-δ, which display distinct physiological functions dependent on their differential ligand activation profiles and tissue distribution [3,4]. PPAR-α is abundantly expressed in skeletal muscles and liver where it increases lipoprotein lipase activity, increases production of enzymes associated with β-oxidation and augments levels of both ApoA-I and high density lipoprotein cholesterol (HDL-C) [5,6].

PPAR-α is also expressed in the brain, where its effects have not been as well characterized. In rodents, Ppar-α has been linked to brain dopamine function, a neurotransmitter system that is a target of some antipsychotic and autism spectrum disorder (ASD) medications. Specifically, Ppar-α activation improves antipsychotic medication adverse event oral tardive dyskinesia [7] and indirectly reduces the activity of dopamine cells in the ventral tegmental area in rodents [8]. In humans, dyslipidemia is more prevalent in individuals with schizophrenia and ASD compared to the general population [9,10], with one of the leading causes of premature death in these patients being CVD [1]. Several genetic association studies report an association between apolipoprotein E (ApoE), which plays an important role in lipoprotein metabolism and CVD, and schizophrenia [11]. Though individuals with schizophrenia and ASD may be at greater risk of dyslipidemia and subsequent CVD, the link between these conditions is not clear.

Here we considered whether the cellular machinery that evolved to modulate energy availability also plays a neurodevelopmental role influencing cognitive behavior.

## 2 Materials and methods

### 2.1 Animals

For all experiments, male mice aged 3–5 months were used. No genotypic differences in body weight were evident at the time of testing. All procedures met the European guidelines for the care and use of laboratory animals (86/609/ECC and 2010/63/UE) and those of the Italian Ministry of Health (D.L. 116/92). Male wild-type and *Ppar*-α *−/−* (*B6.129S4-SvJae-Pparatm1Gonz*) mice previously backcrossed to *C57BL6* mice for 10 generations were bred in house, and the colony was established and maintained by heterozygous crossing. Mice were genotyped as described on the supplier webpage (http://jaxmice.jax.org), with minor modifications. DNA was extracted from tails using the RedExtract kit (Sigma–Aldrich, Italy).

*BTBR T* + *Itpr3tf/J* mice were purchased from Jackson Laboratory and a colony was maintained. All animals were housed in a 12-h light/12-h dark cycle with free access to water and standard laboratory chow diet. For all peripheral treatments, a volume of 10 ml/kg of solution was used.

### 2.2 Stereotaxic surgery

*C57BL6* mice were injected with buprenorphine (0.05 mg/kg^−1^, SC) 30 min before surgery and then anesthetized using a mixture of ketamine and xylazine (100 + 10 mg/kg^−1^, IP). Mice were prepared for surgery, placed in a stereotaxic apparatus (Kopf Instruments) and a small incision was made in the skin above the skull. A drop of a pharmaceutical H_2_O_2_ solution was placed on the skull to increase visualization of bregma and lambda. A small hole was carefully drilled into the skull and a guide cannula (2.0 mm, 26G, Plastics One) aimed at the lateral ventricle (+1.0 mm (lateral), −0.6 mm (posterior) from bregma) was implanted using atlas coordinates (Paxinos and Franklin, 2001) and secured using dental cement. For ICV treatment, 3 μl of vehicle or drug was injected into the lateral ventricle over 1 min, using a 5–10 μl Hamilton syringe connected to a calibrated polyethylene tube. Cannula placement was confirmed at the end of the experiment through injection of methylene blue.

### 2.3 Behavioral assays

#### 2.3.1 Novel Object Recognition (NOR) test

The NOR assay consisted of three testing sessions: a training session followed by two retention trials 15 min and 24 h later. Mice were habituated to the testing arena for two consecutive days before the test. During the training session, two different objects (A and B) were placed in the testing arena. Each animal was allowed to explore the objects for 5 min. The mouse was considered to be exploring the object when the head of the animal was facing the object or the animal was touching or sniffing the object. The total time spent exploring each object was recorded by a trained observer blind to treatment condition and expressed as percentage of total exploration time. In the retention sessions, one identical and one novel object (A and C or D) were used. A mouse was allowed to explore the objects for 5 min, and the time spent exploring each object was recorded. Exploration time was normalized as percentage of total exploration time. Preference for the novel object was considered as successful retention of memory for the familiar object.

#### 2.3.2 Marbles burying test

20 small marbles were arranged in 5 × 4 rows in clean Plexiglas cages with fresh bedding (5 cm deep). Mice were introduced individually to this test arena under dim light and white-noise conditions. After 15 or 30 min, the mouse was removed and the number of unburied marbles was counted. A threshold of 75% coverage was used to determine whether the marbles were buried.

#### 2.3.3 Self-grooming

Mice were manually scored for self-grooming behavior over a period of 10 min under dim light and white-noise conditions.

#### 2.3.4 Spatial learning and memory tests

The Morris Water Maze (MWM) is a circular pool (diameter 170 cm, height 60 cm). The water temperature, 23 ± 1 °C, light intensity, external cues in the room, and water opacity were rigorously reproduced. A transparent Plexiglas non-slippery platform (diameter 10 cm) was immersed under the water surface (about 1.5 cm) during acquisition trails. Swimming was recorded using a camera coupled with video tracking software (Any-maze, Stoelting). Training consisted of 4 swims per day for 9 days, with a 15 min inter-trial interval. Start positions were pseudo-randomly selected and each animal was allowed a 60 s swim to find the platform. Once the mouse reached the platform it was allowed to remain on the platform for 15 s. The latency, expressed as mean ± SEM, was calculated for each training day. A probe test (60 s) was performed 24, 48 and 72 h after the last swim on day 9. The platform was removed, and each animal was allowed a free 60 s swim. The start position for each mouse corresponded to one of two positions remote from the platform location in counterbalanced order. The platform quadrant was termed the target quadrant and the percentage of time spent in the target quadrant was determined.

#### 2.3.5 Reversal learning

A different cohort of mice was trained in the MWM apparatus as described above. On day 10, the platform was moved into a different MWM quadrant (see Figure 2G). The distance swum and the latency to find the new platform position together with the number of crossing on the previous platform position were recorded.

#### 2.3.6 Spontaneous alternation performance

Each mouse, naïve to the apparatus, was placed at the end of one arm in a Y-maze (three arms, 40 cm long, 120 ° separate) and allowed to move freely through the maze during a single 5 min session. The series of arm entries, including possible returns into the same arm, was recorded visually. An alternation was defined as entries into all three arms on consecutive trials. The number of the total possible alternations was therefore the total number of arm entries minus two, and the percentage of alternation was calculated as correct alternations/possible alternations × 100.

#### 2.3.7 NMDA antagonist-induced locomotion

Mice were allowed to explore the arena for 30 min, and their activity was monitored using a video tracking software (Any-maze, Stoelting). After 30 min, recording was paused, mice were injected with vehicle or treatment and immediately returned to the arena, and the video recording re-initiated. MK-801 was administered at a dose of 0.1 mg/kg^−1^; SC and its effect recorded for 120 min. Ketamine was administered at a dose of 20 mg/kg^−1^; IP and its effect recorded for 60 min.

### 2.4 EEG recordings

*Ppar*-α −/− and wild type littermates were chronically implanted with five electrodes under tiletamine/zolazepam (1:1; Zoletil 100^®^; 50 mg/kg, IP; VIRBAC Srl, Milan, Italy) anesthesia. Electrode leads were soldered to a miniature connector, which was head-mounted with cranioplastic cement and mounting screws. Mice were allowed 1 week of recovery and handled twice a day. Mice were attached to a multichannel amplifier (Stellate Harmonie Electroencephalograph; Montreal, Quebec, Canada) by a flexible recording cable and an electric swivel, fixed above the cages, permitting free movements. Mice were acclimated to the recording conditions for 3 days before the experiments. EEG recordings were performed starting at 9:00 a.m. Signals were amplified and conditioned by analog filters (filtering: below 0.5 Hz and above 70 Hz) and subjected to an analog-to-digital conversion with a sampling rate of 300 Hz. Quantitative EEG analysis was performed using the Fast Fourier Transform (FFT) and a commercial software (Stellate Harmonie Electroencephalograph; Montreal, Quebec, Canada). The bipolar signal from each cortical area in both brain hemispheres was processed using FFT and the main spectral components separated. Five main band components were identified in the frequency range between 0.5 and 70 Hz: delta (0.5–3.9 Hz), theta (4.0–7.9 Hz), alpha (8.0–12.9 Hz) and beta (13.0–30 Hz) and gamma (30–70). In both wild type and *Ppar*-α −/− mice, the mean power (in μV^2^) of each of these components was computed by integration of the power spectra in the respective frequency ranges.

### 2.5 Biochemical and histological assays

#### 2.5.1 Preparation of tissue protein extracts and western blot analysis

Dissected brain samples were homogenized on ice-cold lysis buffer (10 mM Tris–HCl, 20 mMpH 7.5, 10 mMNaF, 150 mMNaCl, 1% Nonidet P-40, 1 mM phenylmethylsulphonyl fluoride, 1 mM Na3VO4, leupeptin and trypsin inhibitor 10 μg/ml; 0.25 ml/50 mg tissue). After 1 h, tissue lysates were centrifuged at 14,000 rpm for 15 min at 4 °C. Protein lysates (30–50 μg) were separated on a 10% SDS-polyacrylamide gels. Membranes were probed with primary (mouse anti-Parvalbumin, MAB1572, Millipore, 1:500; rabbit anti-PPAR-alpha, P0369, Sigma–Aldrich, 1:500; mouse anti-β-actin, A5441, Sigma–Aldrich, 1:5000) and the appropriate secondary antibodies (Jackson ImmunoResearch, PA, USA). Immuno-reactive bands were visualized by chemiluminescence using the ImageQuant400 system (GE Healthcare) and the Quantity One Software 4.6.3 (Biorad).

#### 2.5.2 Brain sectioning and immunofluorescence

Mice were deeply anesthetized with pentobarbitone (50 mg/kg^−1^, IP) and transcardially perfused with phosphate buffered saline (PBS) followed by 4% paraformaldehyde (PFA). Following extraction, brains were post fixed overnight in 4% PFA and then transferred to 20% sucrose for cryoprotection. Brains were sectioned on a cryostat at 25 μm and collected in five equal series of adjacent tissue. Free-floating sections were washed in PBS, incubated in blocking buffer (0.5% BSA, 0.5% Triton-X 100 in PBS) for 60 min and then incubated overnight in blocking buffer containing primary antibodies (mouse anti-Parvalbumin, MAB1572, Millipore, 1:1000). Sections were then washed in PBS and incubated for 60 min with secondary antibody (Donkey Anti-Mouse IgG, Alexa Fluor^®^ 488, Abcam, 1:500). Sections were rinsed in PBS, mounted onto glass microscope slides and cover-slipped in an aqueous mounting medium (Vectastain, Vector Laboratories).

### 2.6 Chemicals

All chemicals were from Tocris (Avonmouth, UK), unless otherwise indicated. Fresh drug solutions were prepared immediately before use. GW7647 was administered IP in a vehicle of 0.9% sterile saline with 5% polyethylene glycol (PEG)-400 and 5% Tween 80. WY-14643 was administered in a vehicle of distilled water/5% polyethylene glycol (PEG) 400 and sonicated before injection.

### 2.7 Statistics

Data are expressed as mean ± SEM. Two experimental group comparisons were made using the Student's *t*-test. When more than two experimental groups were present, data were analyzed using one-way ANOVA followed by the Dunnett's post hoc multiple comparison test. Water-maze escape latencies were analyzed over trials using repeated measures ANOVA. For the probe test of MWM, the time spent in the training quadrant was analyzed vs. the chance level (15 s), and for novel object recognition preference for the new object during the retention trail was analyzed vs. the chance level of 50% using Wilcoxon Signed Rank Test. The level of statistical significance was set at *p* < 0.05.

## 3 Results

### 3.1 *Ppar-α* loss of function promotes repetitive behavior and cognitive inflexibility

To investigate the role of *Ppar-α* in behavior, we characterized wild type and *Ppar-α* deficient mice in five behavioral assays: novel object, marble burying, home cage self-grooming, Morris Water Maze (MWM) and Y maze.

**Figure 1:**
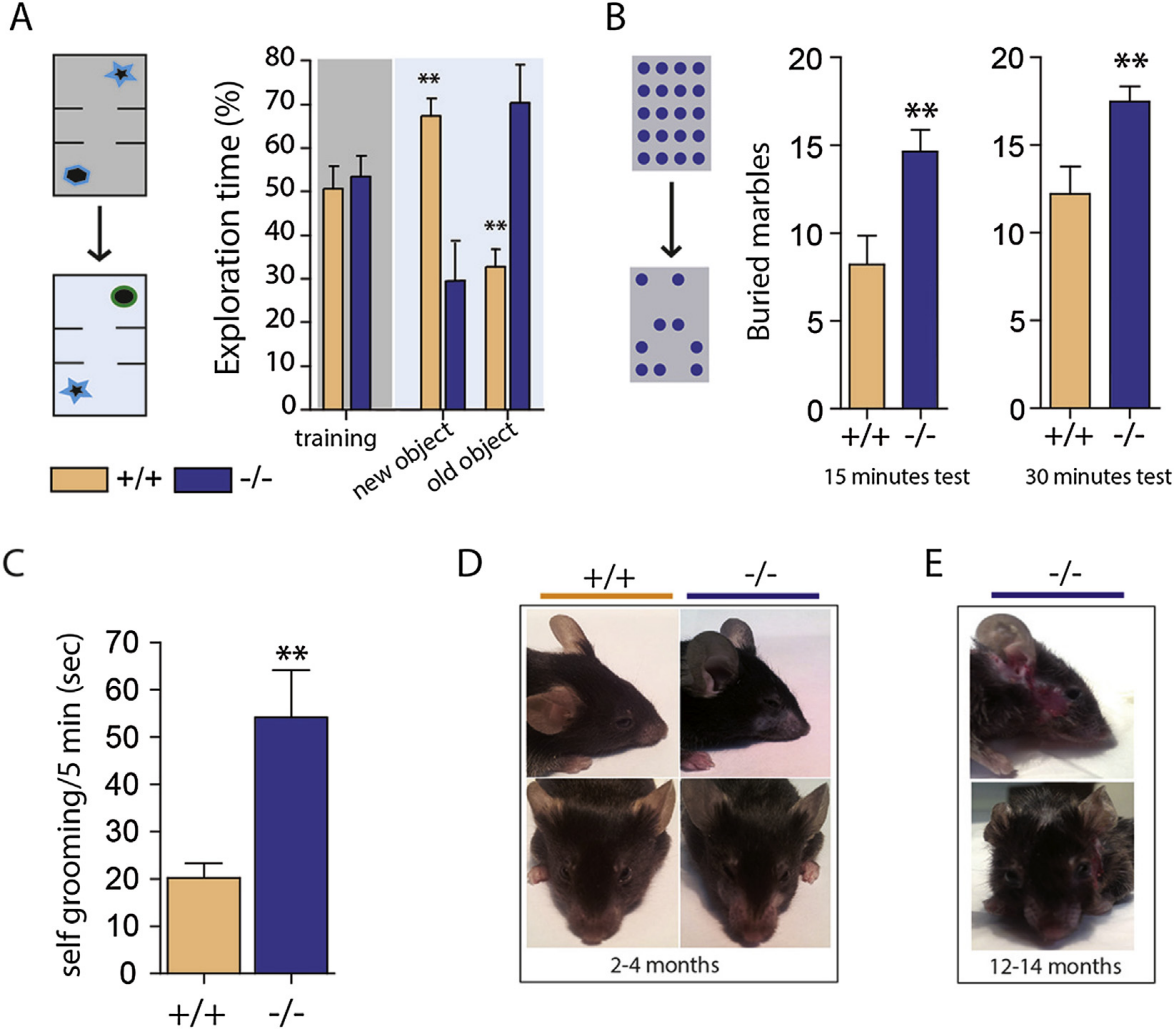
***Ppar-α* −/− mice display enhanced behavioral perseveration and repetition**. **(A)***Ppar-α* −/− mice display enhanced perseveration for the familiar object in the novel object recognition task (n = 8/genotype; **p < 0.01, two-tailed Student's *t*-test). *Ppar-α* −/− mice display enhanced repetitive behavior in the **(B)** marble burying test and **(C)** self-grooming test (3-4 months-old male mice n = 8–9/genotype; **p < 0.01 vs WT, two-tailed Student's *t*-test). **(D)** appearance of facial hair loss in young-adult *Ppar-α −/−* mice. **(E)** skin lesions in middle-aged *Ppar-α* −/− mice.

Mice were first tested for short-term memory through the presentation of a novel object 10 min after the sampling phase (Figure 1A). During the sampling phase of the object recognition test, both wild type and *Ppar-α −/−* mice interacted equally with the two objects. During the short-term test, wild type mice showed a preference for the novel object, whereas *Ppar-α −/−* mice clearly preferred the familiar object (Figure 1A). This observation suggests a conservative behavior of mice lacking *Ppar-α*, and we interpreted the preference for the familiar object during the object recognition task as indicative of a repetitive/perseverative behavioral trait. To further explore this repetitive/perseverative phenotype, mice were assessed in a marble burying test, wherein mice are scored for the number of marbles they bury from the top of the bedding as an index for repetitive/perseverative behavior and compulsion [12]. We observed that *Ppar-α −/−* mice buried significantly more marbles than wild type littermates (Figure 1B), indicative of greater repetitive/perseverative behavior.

The novel object and marble burying assays investigate the response of mice to unfamiliar objects; however, we also considered whether repetitive behavior was also evident in the home cage environment. We scored mice on home cage behavior and observed that *Ppar-α −/−* mice exhibited a significant increase in self-grooming behavior (Figure 1C). Furthermore, by 3 months of age, *Ppar-α* −/− mice developed facial hair loss and sporadic skin lesions (Figure 1D), and the severity of this phenotype increased with age (Figure 1E). This phenomenon did not occur in wild type littermates, even when co-housed with *Ppar-α −/−* mice, and is consistent with behavioral repetition and grooming endophenotypes observed in other genetic mouse models [13–15]. Collectively, these data provide converging evidence of increased repetitive and perseverative behavior as a consequence *Ppar-α* loss of function.

**Figure 2:**
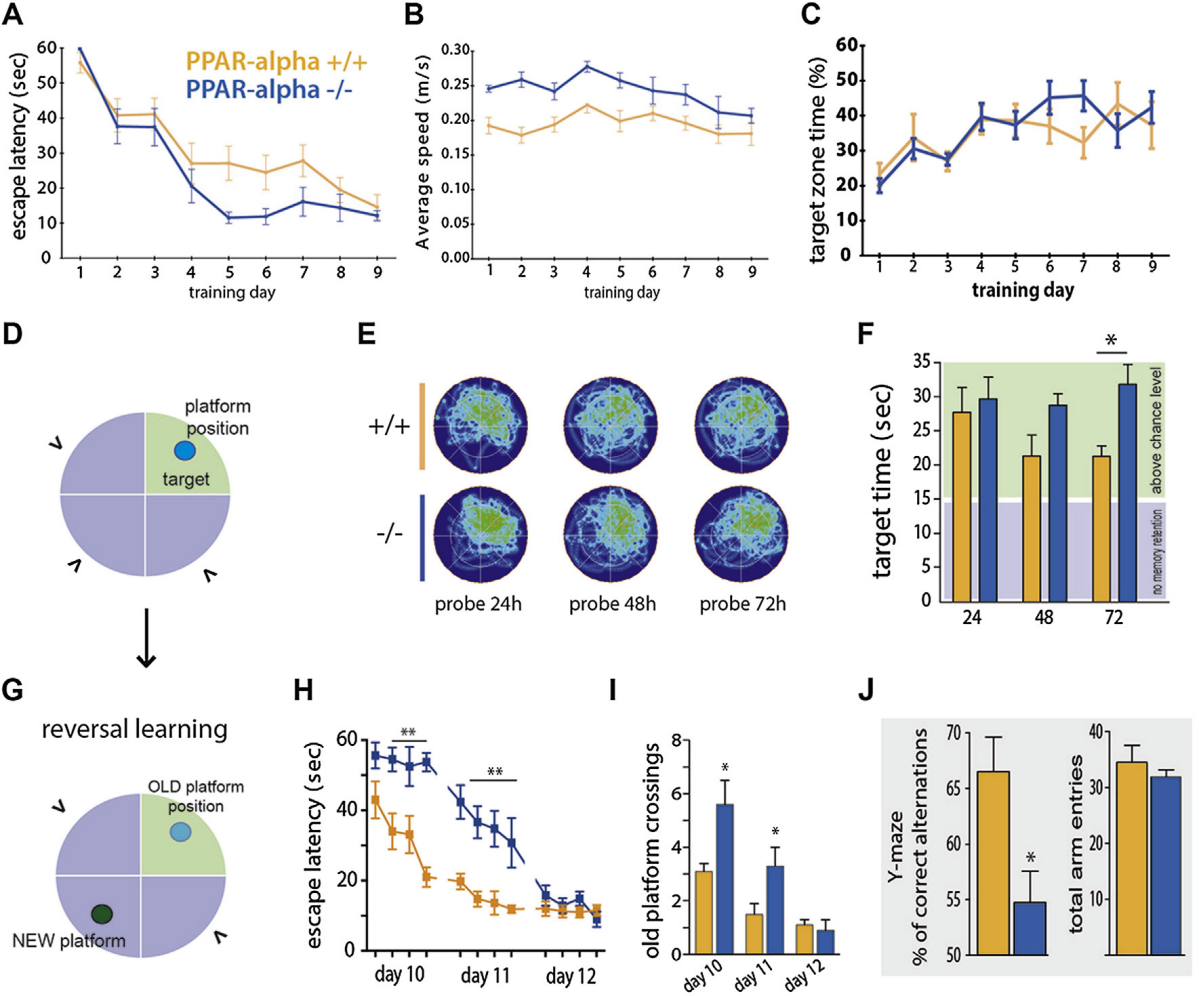
***Ppar-α* −/− mice exhibit impaired *behavioral flexibility***. *Ppar-α* −/− mice display unaffected spatial memory in the Morris Water Maze (MWM) test. **(A)** Escape latency, **(B)** average speed, and **(C)** percentage of time spent in the target quadrant across 9 days of MWM reference platform task shows that *Ppar-α* −/− mice performed similarly to wild-type controls on the spatial hidden platform task. **(D)** Schematic of the MWM (“>” indicates entry points). **(E)** Occupancy plots during repeated MWM probe tests. **(F)** Time spent in the target quadrant during probe tests; although *Ppar-α* −/− mice and wild-type controls spent a significant percentage of time in the target quadrant (*p* < 0.05 *vs* a change level of 15 s; Wilcoxon signed-rank test), during the 72-hours probe test a significant genotype-dependent difference was observed (*p < 0.05, two-tailed Student's *t*-test). *Ppar-α* −/− mice have impaired behavioral flexibility. **(G)** Schematic of the reversal setting of the MWM test. **(H**) Latency to locate the new platform position (tick on the *x-axis* represent test trials; two-way repeated measures ANOVA: effect of genotype; F(1,14) = 30.93, *p* < 0.0001; effect of time: F(11,154) = 34.24, *p* < 0.0001; interaction F(11,154) = 5.18. *p* < 0.0001; followed by Bonferroni *post hoc* test **p < 0.01 *vs* wild type mice at given trials; n = 8). **(I)** Higher number of previous platform position crossings during reversal phase of task compared to controls (*p < 0.05 vs WT, two-tailed Student's *t*-test; n = 8). **(J)** Right, Y-maze spontaneous alternation performances and, left, total numbers of arm maze entries (3-5 months-old male mice n = 10–12; *p < 0.05 vs WT, two-tailed Student's *t*-test).

To confirm that these behavioral effects are not related to global memory impairment, we investigated the impact of genetic *Ppar-α* deficiency on spatial reference memory using the Morris Water Maze (MWM) task (Figure 2A–F). *Ppar-α* −/− mice exhibited a modestly less efficient strategy for locating the hidden platform, with a somewhat shorter escape latency (Figure 2A) and faster swimming speed (Figure 2B). However, the percentage of time spent in the target quadrant increased over test sessions in a manner indistinguishable from wild type littermates (Figure 2C), indicating unaffected learning overall. Furthermore, there was no genotypic difference in memory retention during the MWM test one day after the last learning session (Figure 2E, F), suggesting that *Ppar-α* is not required for spatial memory retention. Moreover, tests performed 48 and 72 h later revealed that both wild type and *Ppar-α −/−* mice spent more time in the target quadrant above the chance level of 25%, indicating appropriate memory retention for the location of the escape platform. However, while wild type mice began to explore other quadrants of the maze seeking different escape possibilities, *Ppar-α* −/− mice spent more time in the target quadrant with minimal exploration in other areas (Figure 2E, F).

We next assessed a new cohort of mice in a reversal-learning task in the MWM by changing the location of the escape platform after mice had learned its position (Figure 2D, G). *Ppar-α* −/− mice manifested a deficit for reversal learning, showing a strong bias toward the platform location they acquired during the training phase, measured as latency to located the new platform position (Figure 2H) and the number of old platform coursing (Figure 2I). These findings were further supported in another behavioral assay, the Y-maze. Again, despite no alterations in reference spatial memory, *Ppar-α* −/− mice displayed a significant impairment in spontaneous alternation when tested in the Y-maze (Figure 2J).

Together, these data suggest that *Ppar-α* is not required for the acquisition of spatial learning and memory. However, *Ppar-α* is required for spatial information processing and cognitive flexibility and loss of functional *Ppar-α* leads to a repetitive/perseverative behavioral phenotype.

### 3.2 *Ppar-α* loss of function promotes an NMDA hypofunction-like condition

**Figure 3:**
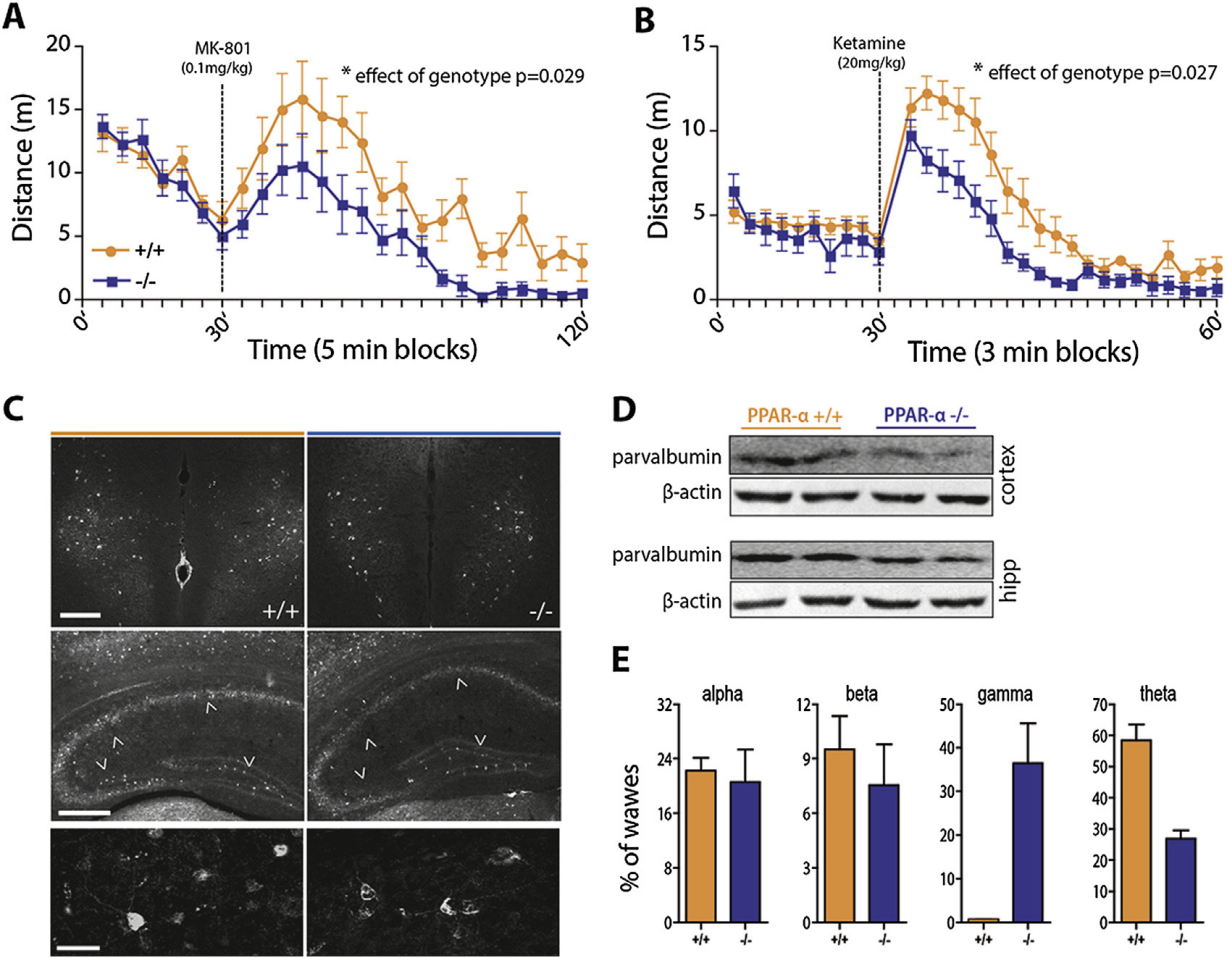
***Ppar-α* loss of function promotes an NMDA hypofunction-like condition**. Non-competitive NMDAR antagonist–induced locomotion is diminished in *Ppar-α −/−* mice. **(A)** Time course of MK-801–induced locomotor activity (0.1 mg kg-1 subcutaneous; two way ANOVA repeated-measures; F(1,360) = 5.81, *p* = 0.0292; n = 7–11). **(B)** Time course of ketamine–induced locomotor activity (20 mg/kg^−1^, IP; two way ANOVA repeated-measures; F(1,348) = 6.29, *p* = 0.0275; n = 7–9). **(C)** Decreased cortical expression of parvalbumin (PV) interneurons in *Ppar-α* null mice; immunofluorescence (PV) staining in the prefrontal infralimbic cortex (top panel; scale bar 200 μm) and hippocampus (middle panel; scale bar 200 μm). Bottom panel – digital magnification of cortical PV interneurons (scale bar 50 μm). **(D)** Western blot of PV expression in frontal cortical and hippocampal explants. **(E)** Alterations of baseline cortical oscillations in awake behaving *Ppar-α −/−* mice. Average relative power in the alpha, beta (top) and gamma and theta (bottom) frequency bands are shown (n = 6 mice/genotype; **p = 0.0022, two-tailed Mann Whitney test).

We next investigated the response of *Ppar-α* −/− mice to a pharmacological model of NMDA hypofunction. Mice were challenged with vehicle or NMDA receptor antagonists that promote behavioral dysinhibition, MK-801 (0.1 mg kg^−1^, SC) or ketamine (20 mg kg^−1^, IP) [16]. *Ppar-α* −/− mice were hypo-responsive to the locomotor effects of both MK-801 (Figure 3A) and ketamine (Figure 3B) in the open field assay. Thus, these results reveal that mice lacking *Ppar-α* are resistant to the behavioral dysinhibitory effect of NMDA antagonists.

We next examined the neural substrates underpinning *Ppar-α* −/− mouse resistance to NMDA antagonists. One possibility is that *Ppar-α* loss of function promotes morphological remodeling of the cortex and/or hippocampus. To investigate this, we examined the anatomical organization of the brain of *Ppar-α* −/− and wild type littermates. No gross abnormalities in brain structure were evident (data not shown). NMDA receptor antagonists induce cortical excitation through a reduction in NMDA receptor function that preferentially decreases the firing rate of fast-spiking parvalbumin positive (PV+) GABA-ergic interneurons [17,18], resulting in dysinhibition of pyramidal neurons. The net effect is cortical excitation [19,20]. Using both immunofluorescence staining and western blotting techniques, we investigated the expression profile of parvalbumin interneurons in the frontal cortex and hippocampus. We observed that *Ppar-α* −/− mice displayed a reduction of PV + interneurons in frontal cortex and hippocampus (Figure 3C, D), and that remaining PV + interneurons appeared dystrophic (Figure 3C, bottom panel). These data provide an anatomical explanation for the hypo-responsiveness of *Ppar-α* −/− mice to NMDA antagonists.

To further examine whether this anatomical impairment impacts brain function, we performed EEG. PV + interneurons are essential for synchronization of neural activity, and their impairment causes EEG alterations [21–23]. EEG recordings in freely moving mice revealed a genotype-dependent difference between *Ppar-α* −/− mice and wild type littermates. Deletion of *Ppar-α* caused an abnormal increase in gamma waves, while theta frequency was lower compared to wild type mice (Figure 3E). Together, these data reveal that *Ppar-α* loss of function impacts the structural morphology and functional integrity of the cortex and hippocampus and the synchronization of neural activity.

### 3.3 Antipsychotic medication improves repetitive behavior in *Ppar-α* −/− mice

**Figure 4:**
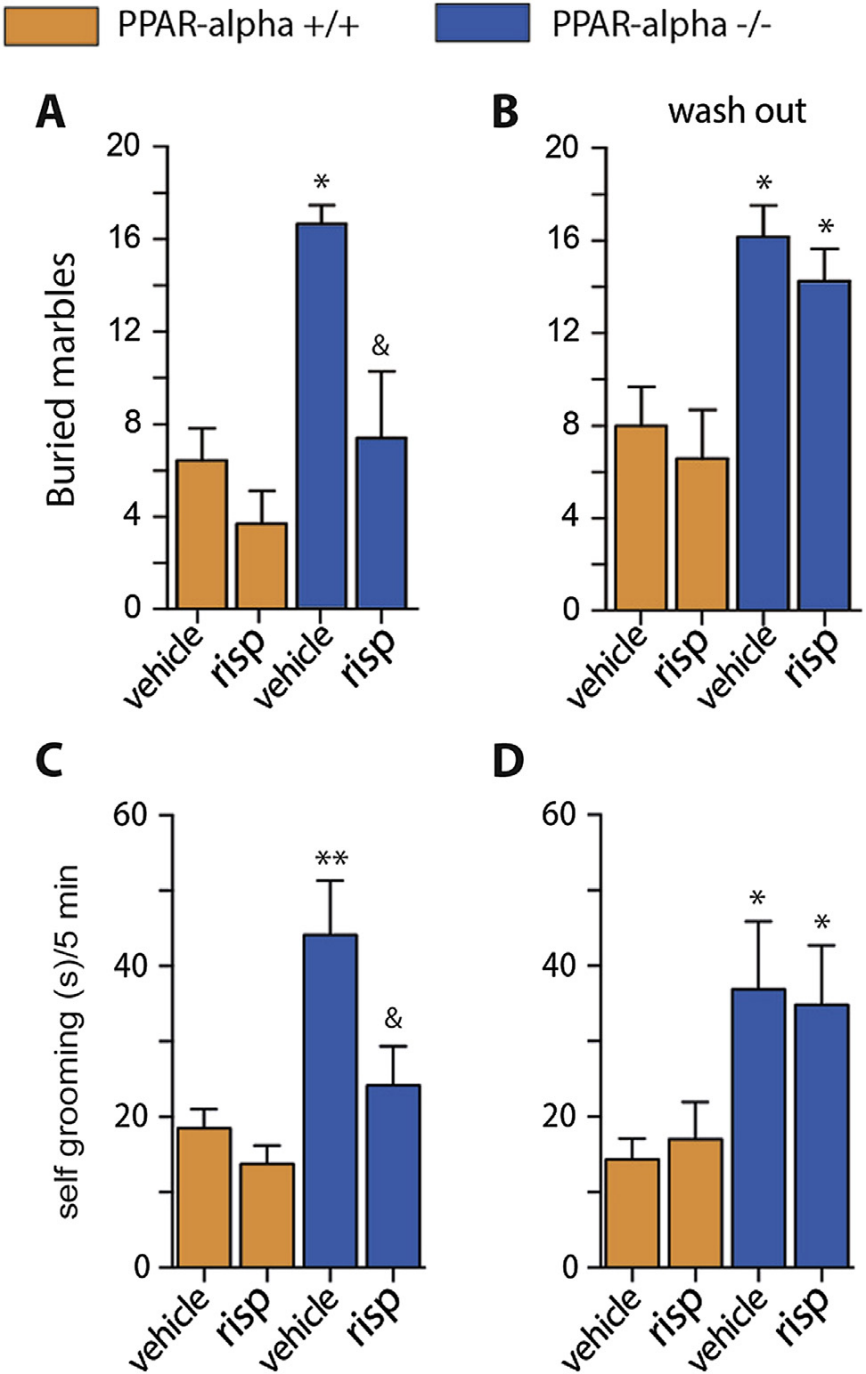
**Risperidone improves repetitive behavior in *Ppar-α* null mice**. Effect of 7–10 days treatment with risperidone (0.01 mg/kg^−1^, IP) on marble burying behavior **(A)** and self-grooming **(C)**. Re-appearance of the increased marble burying **(B)** and self-grooming behaviors **(D)** one week after treatment's suspension (3 months-old male mice n = 8–12; *p < 0.05 and **p < 0.01 *vs* wild type mice, &*p* < 0.05 *vs Ppar-α* −/− mice treated with vehicle; one-way ANOVA, followed by Tukey's multiple comparisons test).

To investigate whether the behavioral alterations observed in *Ppar-α* −/− are ameliorated by antipsychotic medication, wild type and *Ppar-α* −/− littermates were treated with risperidone or vehicle for 7–10 days and were then assessed in the marble burying assay, self-grooming in the home cage and MWM tasks. Risperidone (0.01 mg/kg, IP) normalized the behavior of *Ppar-α* −/− mice in the marble burying test (Figure 4A), and it reversed the elevated self-grooming behavior (Figure 4C). It did not, however, correct the reversal-learning deficit in the MWM (data not shown). Mice were retested 7 days after risperidone was withdrawn, and both marble burying and self-grooming behavior returned to levels similar to those observed in unmedicated mice (Figure 4B, D). Thus, *Ppar-α* −/− mice exhibit an improved behavioral profile when treated with an antipsychotic medication.

### 3.4 Pharmacological activation of PPAR-α reduces NMDAR antagonist–induced locomotion and reduces repetitive behavior in *BTBR T* + *tf/J* mice

**Figure 5:**
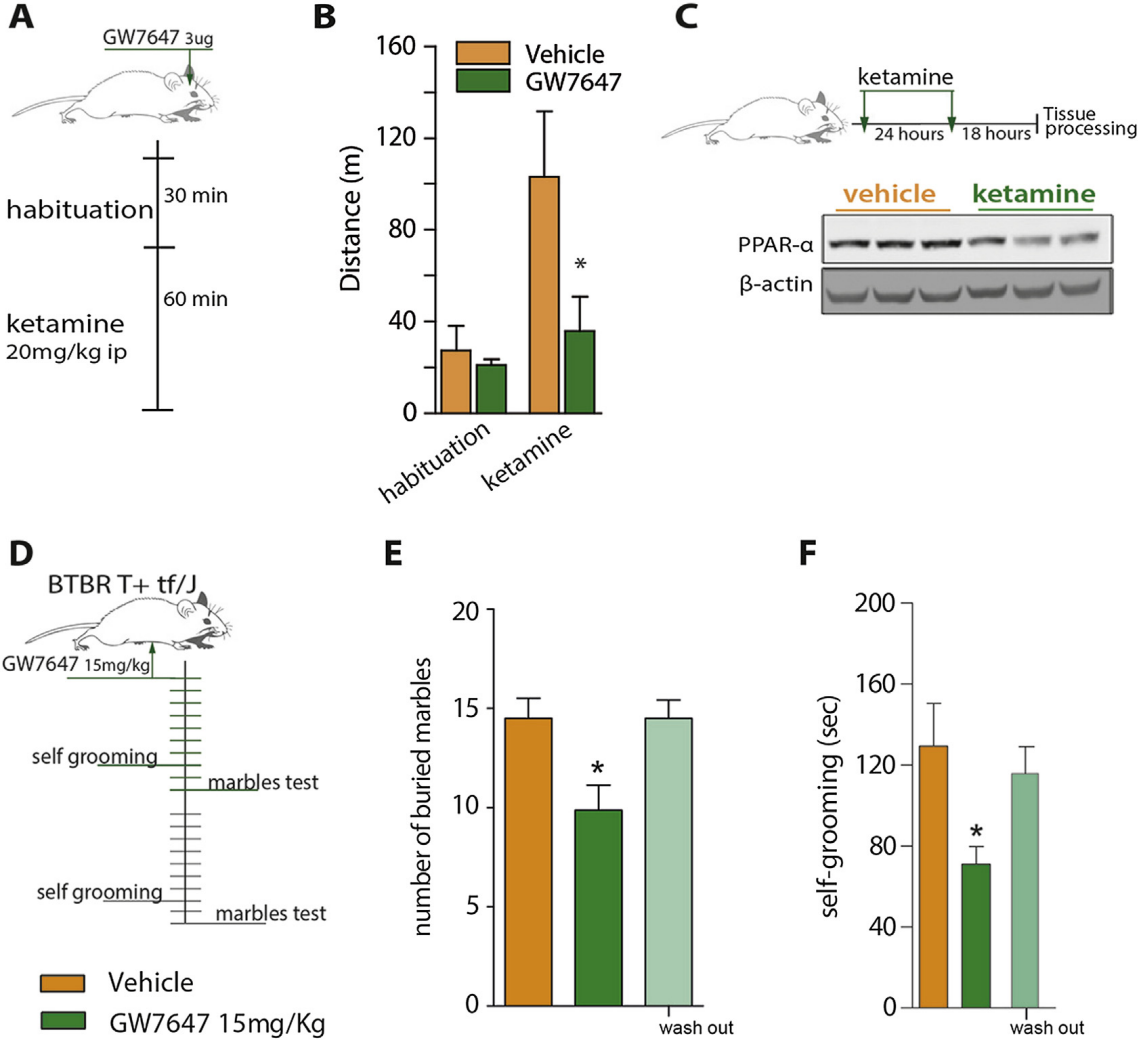
**Pharmacological activation of Ppar-α reduces NMDAR antagonist–induced locomotion and reduces repetitive behavior in BTBR T+ tf/J mice**. A single brain administration of a selective PPAR*-α* agonist reduces ketamine–induced locomotion. **(A)** Treatment schematic: the PPAR*-α* agonist, GW767 (3ug) administered into the lateral brain ventricle (ICV) 30 min before ketamine (20 mg kg-1, IP). **(B)** The cumulative locomotion during the habituation and after ketamine injection (n = 6–7; *p < 0.05 *vs* vehicle-ketamine group; two-tailed Student's *t*-test). **(C)** Down-regulation of cortical PPAR*-α* following repeated ketamine injections in wild type mice (n = 3). Treatment with a PPAR*-α* agonist ameliorates behavioral repetition in a genetic model of ASD – the BTBR T+ tf/J mice (male, 14 weeks of age). **(D)** Treatment schematic: GW767 (15 mg kg-1, IP) treatment for 7–10 days followed by a wash out week. **(E)** Effect of GW767 treatment on the marbles burying and **(F)** self-grooming behavior (n = 8–10; *p < 0.05 *vs* vehicle treated mice, &*p* < 0.05 *vs* GW767-treated; one-way ANOVA, followed by Tukey's multiple comparisons test).

To investigate the therapeutic application of our findings, we next probed the consequence of pharmacological activation of PPAR-α in preclinical models of schizophrenia and ASD. We hypothesized that the activation of brain PPAR-α would be sufficient to attenuate the psychomimetic action of the NMDA receptor antagonist ketamine. Ketamine was selected because it induces symptoms associated with schizophrenia in healthy human volunteers and as such is used as a pharmacological model of schizophrenia-like behavior. Wild type mice were pretreated with the selective PPAR-*α* agonist GW7647 (3 μg, ICV) and treated with ketamine (20 mg kg^−1^, IP). PPAR-α agonist treatment reduced ketamine-induced hyperlocomotion in the open field assay (Figure 5A, B). Further underscoring a link between Ppar-α brain expression and cortical dysinhibition, we observed that during a period of sustained cortical dysinhibition, wild type mice displayed lower levels of cortical PPAR-α when treated with two injections of ketamine (Figure 5C). Therefore, these data reveal that activation of PPAR-α is effective in improving the psychomimetic effect of ketamine.

We next assessed the effect of PPAR-*α* agonist GW7647 on behavior in *BTBR T* + *tf/J* (BTBR) mice, a mouse model that displays reliable face validity for ASD [24,25], including high rate of repetitive behavior [26]. BTBR mice were treated with GW7647 (15 mg/kg, IP) or vehicle for 10 days and subsequently scored for spontaneous marble burying and self-grooming behaviors (Figure 5D). PPAR-α activation significantly reduced the number of buried marbles (Figure 5E) and improved the spontaneous grooming behavior (Figure 5F) in BTBR mice. Seven days after GW7647 treatment was withdrawn, behaviors returned to levels similar to those observed in untreated BTBR mice. These data suggest that daily treatment with a PPAR-α agonist improves repetitive behavior in BTBR mice.

## 4 Discussion

PPAR-α is best characterized for its contribution to the regulation of lipid homeostasis and its utility as a target for the treatment of dyslipidemia. However, little is known about its function within the brain. Here we reveal an unexpected role for *Ppar-α* in cognitive behavior in mice.

Specifically, we report that genetic inactivation of *Ppar-α* resembles a behavioral and cognitive phenotype consistent with preclinical models of schizophrenia and ASD. In an effort to elucidate the mechanism through which this phenotype is mediated, we analyzed the brain. We observed that the genetic prevention of Ppar-α activity produced a reduction in cortical PV + GABAergic interneurons, consistent with post-mortem analyses of brains from patients with schizophrenia [27–30]. We also report that the behavioral profile of *Ppar-α* null mice was improved with antipsychotic risperidone treatment. Thus, *Ppar-α* deficient mice may represent a new preclinical model to investigate the etiology and/or treatment of schizophrenia. The behavioral profile of *Ppar-α* null mice also shows similarities with ASD mouse model BTBR, which displayed an improved repetitive behavior with PPAR-α agonist treatment. Furthermore, risperidone is also used to alleviate hyperactivity, self-injurious, and repetitive behavior symptoms in humans suffering with ASD [31,32]. Together, these results highlight PPAR-α as a potential point of commonality between schizophrenia and ASD worthy of further investigation.

Considering that repetitive behaviors can arise from a disruption in the direct cortico-striatal circuit, it is possible that PPAR-α plays an instrumental role in the organization and orchestration of PV + interneuron-pyramidal neuron cortical microcircuitry, and the absence of this regulation contributes to a net increase in cortical firing and output onto striatal structures. Supporting this possibility, GABAergic interneurons are crucial for synchronization of network activity, *Ppar-α* −/− mice exhibit abnormal EEG waves, and *Ppar-α* −/− mice are resistant to the behavioral dysinhibition caused by the administration of NMDA receptor antagonists, which inhibit the activity of cortical PV + interneurons.

Given our observation that PPAR-α agonist treatment improves behavior in a pharmacological model of psychosis (ketamine) and a mouse model that displays face validity for ASD (BTBR) [24–26], our research suggests that patients with schizophrenia and ASD co-prescribed fibrates to improve dyslipidemia may show a greater benefit in cognitive symptom amelioration. This possibility warrants further investigation in patient populations. Moreover, it is possible that at least a subset of these patients may receive direct therapeutic benefit from fibrates. If this were the case, it would have the added benefit of overcoming the metabolic disturbance associated with many current antipsychotic medications [33–37]. Of interest, loss of function of *Ppar-α* results in middle age-onset obesity/weight gain in mice [38]. Thus, increasing the activity of PPAR-α with compounds such as fibrates in patients may serve a further metabolic-protective role.

In conclusion, our findings disclose a previously unknown role for Ppar-α in cognitive function in mice. In addition to highlighting a neurological phenotype resulting from the loss of function of *Ppar-α*, our findings also suggest that this receptor may represent a target for the pharmacological amelioration of neurological conditions associated with behavioral perseveration/repetition. This is a particularly attractive prospect given that naturally occurring and synthetic PPAR-α agonists are currently used in clinical practice.
